# Peer Victimization and Depressive Symptoms Among Rural-to-Urban Migrant Children in China: The Protective Role of Resilience

**DOI:** 10.3389/fpsyg.2016.01542

**Published:** 2016-10-04

**Authors:** Zhi Ye, Lihua Chen, Sayward E. Harrison, Haiying Guo, Xiaoming Li, Danhua Lin

**Affiliations:** ^1^Institute of Developmental Psychology, Beijing Normal UniversityBeijing, China; ^2^Department of Health Promotion, Education, and Behavior, University of South Carolina, ColumbiaSC, USA

**Keywords:** peer victimization, resilience, depressive symptoms, migrant children, moderate effect

## Abstract

Peer victimization can have a profound effect on children’s wellbeing and is a known risk factor for depression in childhood. Migrant children experience peer victimization at higher rates than non-migrant peers; however, limited research has examined psychological factors that may serve to reduce depression risk for this group. In particular, no studies have yet investigated whether resilience, including personal characteristics, and a strong social support network, may moderate the relationship between peer victimization and depressive symptoms for migrant children. This study utilized a latent interaction model to examine the effect of resilience on the relationship between peer victimization and depressive symptoms among 721 rural-to-urban migrant children in Beijing, China. Results indicated that peer victimization was positively associated with depressive symptoms. Resilience was found to be a protective factor for depressive symptoms and also mitigated the effects of peer victimization on depressive symptoms. Exploratory analyses suggest that enrollment in private migrant schools may be linked with poorer psychosocial outcomes for Chinese migrant children. Strengthening the internal resilience and social supports for all migrant children may be an effective strategy to lower their risk for depression. Implications for intervention are discussed.

## Introduction

Being victimized has negative and far-reaching effects on children and is a risk factor for internalizing and externalizing problems, poor school achievement, and even suicide (Sentse et al., 2013). In this study, we examine the relationships among peer victimization, resilience, and depressive symptoms for children who migrate with their parents from rural areas to Beijing, China. First, we investigate whether peer victimization and resilience are significant predictors of depressive symptoms. Next, we explore the protective effect of resilience on the relationship between peer victimization and depressive symptoms.

Since the late 1970s, China has experienced increases in economic disparity and rapid urbanization that have resulted in an unprecedented growth of rural-to-urban migration. This migration typically follows a pattern of movement from the western and central inlands of China (e.g., Sichuan, Henan, Anhui, and Hunan provinces) to eastern coastal locations (i.e., Beijing, Shanghai, and Guangdong provinces). Migrants move to urban centers in search of better jobs, increased educational opportunity, and improved living conditions. However, they frequently find themselves residing in unsanitary, overcrowded dormitories or in shared accommodations and working in physically demanding jobs such as manual labor, factory work, or service work where they are paid low wages (Wong and Song, 2008). Adults who migrate to urban centers often bring their children with them (Chen et al., 2009). Thus the number of Chinese migrant children who are residing in urban areas has substantially increased in recent years (Hu et al., 2014). In 2013, approximately 35.8 million migrant children and adolescents were estimated to reside in China, representing a 41.4% increase since 2005 and accounting for 12.8% of the current child population in China (Mao and Zhao, 2012; All-China Women’s Federation, 2013; Hu et al., 2014). Beijing is currently estimated to have approximately 366,000 migrant primary-school-aged children, accounting for about 44.6% of all students enrolled in primary schools (Beijing Municipal Bureau of Statistics and Nbs Survey Office in Beijing, 2015).

Children who migrate to host cities with parents are likely to encounter unique challenges when compared to their non-migrant counterparts. Under China’s Household Registration System (i.e., *hukou* system), Chinese citizens are categorized into either an urban or rural residency status. Migrant families maintain their rural residential status after relocating to an urban area. Because public school enrollment is contingent upon local legal residency, children of migrant workers are frequently denied enrollment in local public schools. While some parents are able to gain entry for their children into public schools through paying special fees or other means, most must send their children to inexpensive private migrant schools that are often constructed and staffed by migrant workers themselves (Chen and Feng, 2013).

In Beijing, these private migrant schools are typically located within migrant enclaves and have limited access to educational resources (Chen et al., 2009). Most private migrant schools do not receive government subsidies as they operate informally and are typically staffed by under-qualified teachers, offer very limited curricula, and operate with poor facilities (Goodburn, 2009; Chen and Feng, 2013). Moreover, even migrant children who are able to enroll in public schools face numerous challenges; most are still ineligible to take middle or high school entrance examinations in Beijing and must return to their hometowns if they wish to seek further education (Wu et al., 2012). Lack of legal residency also means that Chinese migrant children residing in urban areas frequently have limited access to health care and other social services (Liang and Chen, 2007; Zhang et al., 2010). These sociocultural systems interact with and shape children’s behavioral and psychological health (Bronfenbrenner, 1979, 1989) and pose particular challenges for migrant children.

Within this context of marginalization and limited resources, migrant children must navigate the complex processes of acculturation and adaptation. The acculturation process has been theorized to consist of two distinct experiences: (1) sociocultural adjustment to a new set of customs and cultural expectations, and (2) psychological changes one’s cultural identity, attitudes, and social behaviors flex and bend (Berry, 2003; Phinney, 2003). This process frequently yields “acculturation stress” (Berry et al., 2006) that challenges multiple domains of functioning including emotional wellbeing, social functioning, and adaptive skills (Wang and Mesman, 2015). Within their new environments, migrant children are often perceived to be “outsiders” and may have difficulty adapting into their new urban society (Hu et al., 2014).

In fact, the impact of the migrant experience on the psychological wellbeing of Chinese children has been extensively studied in the past decade, with results suggesting that compared with non-migrant urban peers, migrant children suffer from a higher prevalence of mental health problems, including depression, social anxiety, and problem behavior (Li et al., 2008; Lin et al., 2009). Both residential and school mobility represent significant ecological transitions for children, and mobility is a known risk factor that may negatively impact the ability of children to develop positive relationships and achieve academic success, especially for low-income children or children living in non-traditional family structures (Tucker and Long, 1998; Temple and Reynolds, 1999). Due to their status as “newcomers” in their social environments (e.g., school, neighborhood), migrant children are often discriminated against in urban areas because they are believed to have undesirable characteristics (e.g., low socioeconomic status, accent, undesirable appearance, unfamiliar customs, or behaviors) that mark them as different and cause them to be devalued, rejected, excluded, or victimized by peers (Guo, 2007; Chen et al., 2014). Tan (2010) investigated 758 migrant children from two cities in China and found that approximately 21.8% of migrant children were involved in bullying, significantly higher than the prevalence of 14.9% among the general child population in China (Zhang, 2002).

Peer victimization is defined as “the experience among children of being a target of the aggressive behavior of other children” (Hawker and Boulton, 2000). It can take many forms, including physical harassment, verbal harassment, relational aggression, and property-related victimization. While such incidents are relatively common during childhood and early adolescence, peer victimization poses significant threats to children’s wellbeing, especially when chronic (Mynard and Joseph, 2000). Previous research has consistently reported that, compared with non-victimized peers, children who experience peer victimization are at-risk for a number of psychological difficulties, including problem behavior, anxiety, loneliness, poor life satisfaction, and low self-esteem (Diler et al., 2003; Gu et al., 2010; Liu et al., 2014). Operating in a negative feedback loop, such emotional difficulties can contribute to increased levels of acculturation stress and can cause further isolation for affected children and families.

Among the various types of internalizing problems, peer victimization is most strongly associated with depressive symptoms in childhood, especially during middle childhood (Hawker and Boulton, 2000; Ghoul et al., 2013; Roeder et al., 2014). There is evidence that the experience of peer victimization predicts the development of negative cognitions and reductions in positive cognitions, and that this, in turn, contributes to the development of depression (Sinclair et al., 2012). Both cross-sectional and longitudinal studies have suggested a strong association between peer victimization and depression (Seals and Young, 2003; Hong and Espelage, 2012; Cole et al., 2014). Being an occasional target of bullying has been found to be associated with risk of depressive symptoms, while frequent exposure to victimization is linked with risk for clinical depression (Klomek et al., 2007). Thus peer victimization may display a dose-response relationship with negative mental health outcomes, with higher doses of victimization yielding higher levels of mental health problems (Evans et al., 2014). Longitudinal studies have also found peer victimization to be a significant and positive predictor of depressive symptoms among children over time (Bond et al., 2001; Snyder et al., 2003; Schwartz et al., 2005). For instance, being a victim of school bullying at the age of 15 predicted higher levels of self-reported depressive symptoms throughout later adolescence and early adulthood, suggesting that the effects of bullying are serious and long lasting (Due et al., 2009). The link between victimization and depression has also been documented for Chinese children. A recent study investigated associations between peer victimization and psychosocial adjustment among 1,767 Chinese children, with results indicating that peer victimization significantly predicted the experience of depressive symptoms (Ji et al., 2011). However, data is limited regarding the relationship between peer victimization and depressive symptoms for rural-to-urban migrant children in China.

Due to the heightened risks faced by migrant children, there is a need to identify protective factors that can help this population adapt to their new environments in a successful manner and manage the accompanying psychological challenges that acculturation encompasses. Such protective mechanisms can broadly be characterized as facets of “resilience.” While resilience has no universally accepted definition, psychological resilience is typically thought to be a dynamic process that allows an individual to positively adapt in the face of adversity (Luther et al., 2000; Li et al., 2015). Resilience thus serves as a protective process that can buffer the deleterious effects of individual and/or contextual risk factors (Fincham et al., 2009; Masten and Narayan, 2012). In developmental literatures, the process of resilience enables an individual to adapt in the face of risk, trauma and other aversive life circumstances, and has typically been conceptualized as operating at three broad levels: at the community level (e.g., neighborhood characteristics, social supports), at the family level (e.g., parental warmth, close caregiver relationship), and at the child level (e.g., hardy personality, intelligence) (Werner and Smith, 1982; Garmezy, 1985). The protective ability of individual characteristics has been well examined among children exposed to various adversities, and multiple protective factors have been identified including a hardy personality, strong self-efficacy, hope for the future, self-enhancing cognitions, positive coping skills, and positive emotion (Bonanno, 2004).

Multiple studies have used moderation analysis to examine the ability of resilience to confer protection against the adverse effects of contextual risk. Wingo et al. (2010) found that resilience moderated depressive symptom severity among 792 American adults exposed to childhood trauma. Specifically, given similar levels of trauma exposure, individuals with high resilience displayed significantly lower depressive symptoms than those with low resilience (Wingo et al., 2010). Similarly, Campbell-Sills et al. (2006) examined the psychological wellbeing of 132 American college students and also found that resilience was a moderator in the relationship between emotional neglect in childhood and current psychiatric symptoms. While resilience has garnered a broad literature base through investigations with Western populations, efforts to investigate resilience with Eastern populations are more limited. Lee and Cranford (2008) found that resilience played a protective role in buffering the negative effects of risk factors on depressive symptoms among children of alcoholic parents in Korea. Peng et al. (2012) investigated 2069 Chinese medical students and reported that resilience moderated the relationship between negative life events and mental health problems. In addition, resilience was found to moderate the effects of negative events on adolescent depressive symptoms for 2,250 Chinese children and adolescents 6 months after experiencing the devastating Wenchuan earthquake (Zhu et al., 2012). While such studies have sought to identify the protective influence of resilience on depressive symptoms among at-risk children and adolescents, little work has yet examined the ability of resilience to moderate the relationship between peer victimization and depressive symptoms, particularly among rural-to-urban migrant children in China.

The current study attempts to fill these gaps by investigating the following research questions: (1) Is peer victimization a significant predictor of depressive symptoms among rural-to-urban migrant children in China? (2) Does resilience in migrant children moderate the relationship between peer victimization and depressive symptoms? Based on previous work, we hypothesized that peer victimization would be a significant predictor of migrant children’s level of depressive symptoms. Furthermore, we hypothesized that resilience would moderate the relationship between peer victimization and depressive symptoms, such that the negative effects of peer victimization on depressive symptoms would be weaker among migrant children with higher levels of resilience.

## Materials and Methods

### Participants and Procedure

In the current study, migrant children from the 4th, 5th and 6th grades of three primary schools (i.e., two traditional public schools and one private migrant school) in the Daxing district of Beijing were recruited for participation. The research team initially worked with school principals to generate lists of eligible migrant children. The eligibility criteria for migrant children included the following: (1) no household register (i.e., *hukou*) in Beijing and (2) temporarily living with parents who have migrated to Beijing for employment for more than 3 months. A total of 721 adolescents were recruited for the study. The sample included 384 (53.3%) migrant adolescents attending traditional public school and 337 (46.7%) migrant children attending a private migrant school. Of the 721 participants, 277(38.4%) were girls and 444 (61.6%) were boys. The grade distribution was as follows: 251 (34.8%) were enrolled in 4th grade, 221 (30.7%) were enrolled in 5th grade and 246 (34.1%) were enrolled in 6th grade. Four of them did not report their grade. The mean age estimate for children enrolled was 10.22, *SD* = 1.02 (The Ministry of Education of the People’s Republic of China, 2014).

After acquiring permission from school principals to conduct the survey in their schools, eligible children who agreed to participate were asked to complete a battery of self-administered questionnaires. Children completed the questionnaires in a classroom, and trained interviewers (i.e., 15 psychology graduate students) circulated among them to answer any questions or provide necessary clarification. Each child received an age-appropriate gift (i.e., toy or school supplies) at the completion of the survey as a token of appreciation. All study procedures were approved by the Institutional Review Board (IRB) at Beijing Normal University.

### Measures

#### Peer Victimization

Peer victimization was measured by creating a modified multidimensional scale derived from the Chinese version of the *Multidimensional Peer Victimization Scale* (Zhang et al., 2009), the *Children’s Peer Victimization Scale* (Dong, 2007), and the *Social Experience Questionnaire-Self Report* (Crick and Bigbee, 1998). The modified scale contains 18 items that assessed physical victimization (three items; e.g., “Other kids hurt me physically in some way”), relational victimization (seven items; e.g., “Some kids tried to get me into trouble with my friends”), verbal victimization (five items, e.g., “Other kids swore at me”) and property victimization (three items; e.g., “Some kids tried to break or steal something of mine”). Each item was rated on a 4-point scale, ranging from 1 (never) to 4 (a lot). Confirmatory factor analysis indicated that the four-factor model demonstrated a good fit to the data: *χ^2^/df* = 1.64, CFI = 0.94, TLI = 0.93, RMSEA = 0.05. The Cronbach’s *α* coefficients were 0.80, 0.89, 0.82, 0.66, respectively, for subscales and 0.93 for the full scale.

#### Resilience

The 27-item *Resilience Scale for Chinese Adolescents* (RSCA, Hu and Gan, 2008) was used to evaluate children’s resilience. The scale measures the ability of an adolescent to cope with stress and adversity and has been used widely in research on Chinese children’s resilience (Wang et al., 2014; Zhang and Tian, 2014). The questionnaire contains two subscales that measure personal assets and social resources. The personal assets subscale measures an individual’s internal capacity to cope with difficulties, while the social resources subscales measure an individual’s perceived support from peer and family members and the competency for support seeking when encountering adversity. The following are examples of items: “The experience of frustration made me more mature” and “I believe adversity can inspire people.” Responses to the items are given on a 5-point Likert scale (1 = strongly disagree, 2 = disagree most of the time, 3 = agree sometimes, 4 = agree most of time, 5 = strongly agree), with higher scores indicating higher levels of resilience. In the current study, the Cronbach’s alpha coefficients of the personal assets and social resources subscales were 0.79 and 0.78, respectively, and the full scale’s Cronbach’s alpha was 0.86, indicating good internal consistency.

#### Depressive Symptoms

Depressive symptoms were assessed with the self-reported *Center of Epidemiological Studies Depression Scale for Children* (CES-DC), developed by Fendrich et al. (1990). CES-DC contains 20 items and measures symptoms associated with depression. Children rate each item with regard to how frequently they have experienced the symptom in the previous week. Sample items from the measure include: “I did not feel like eating and my appetite was poor” and “I felt sad.” The response options rated each item on a 4-point scale ranging from 0 (not at all) to 3 (a lot) with higher scores indicating higher levels of depressive symptoms. The scale displayed good internal consistency (*α* = 0.83) for the sample in the current study. In order to meet standards for constructing a latent variable interaction model, item parceling was used to create a latent variable for depressive symptoms. In this study, the Cronbach’s *α* of the three subscales were 0.54, 0.57, and 0.65, while the internal consistency estimate of the full scale was 0.83.

### Statistical Analysis

Statistical analyses were performed using SPSS 22.0 and Mplus7.1 (Muthén and Muthén, 1998–2012). First, descriptive statistics, including means, standard deviations, and bivariate correlations, were calculated by group (i.e., private migrant school, public school). Second, hypotheses were tested using structural equation modeling to assess the latent variable interaction effect estimation of peer victimization and resilience on depressive symptoms. The interaction model was tested using the latent variable interaction approach described by Klein and Moosbrugger (2000). A two-step approach was used to model the latent variable interaction. Firstly, peer victimization and resilience were used to jointly predict depressive symptoms (Model 1). Only linear effects of the predictors were considered in this conventional structural equation model. Next, the latent moderated structural equation method was used to examine the latent interaction effect between peer victimization and resilience on depressive symptoms (Model 2).

The technique of item parceling was employed to construct the measurement model of depression for two reasons. First, compared to item-level data, parceling could provide more stable factor solutions (Little et al., 2002). Second, item parceling can reduce the estimate parameters and the likelihood of residuals being correlated, which can further reduce the measurement error of the model. Three parcels for depression were created based on the method of item-to-construct balance (Landis et al., 2000). To do this, CES-DC was subjected to a factor analysis in which a single-factor solution was specified. Then, the highest and lowest factor loadings were combined as a set. The construct for depression was unidimensional in nature, which indicated that some of the pitfalls of item parceling (e.g., misrepresentation of the factor model) were avoided in this study (Chang and Edwards, 2015). Item parceling was not used for the measures of peer victimization and resilience because they are both multidimensional constructs that are well represented by the original subscales.

To enhance the interpretability of results, we used the Robust Maximum Likelihood estimator as it does not require normal distribution of variables, and standardized all indicators before running the analyses. In addition, we used the Root Mean Square Error of Approximation (RMSEA; 0.05 or below indicating excellent fit, 0.05–0.08 reflecting an acceptable fit), Comparative Fit Index (CFI; 0.95 or above indicating excellent fit, 0.90–0.95 reflecting an acceptable fit), and Tucker-Lewis Index (TLI; 0.95 or above indicating excellent fit, 0.90–0.95 reflecting an acceptable fit) to test model fit for all models. Gender and grade were controlled as potential confounders. However, none of these variables were found to significantly predict the outcome, so they were dropped from the analysis.

Multiple-group analysis was also employed in an exploratory analysis to test for statistically significant differences in the structural parameters across groups (i.e., children enrolled in private migrant school vs. public school).

## Results

### Descriptive Statistics by School Group and Correlates of Peer Victimization, Resilience, and Depressive Symptoms

As shown in **Table 1**, migrant children currently enrolled in the private migrant school reported significantly higher levels of victimization, more depressive symptoms, and lower level of resilience than migrant children enrolled in the traditional public schools.

**Table 1:** Descriptive statistics for peer victimization, resilience and depressive symptoms.

	Overall	Migrant childrenin public school	Migrant children inprivate migrant school	*t*	*p*
*N* (%)	721 (100%)	384 (53.3%)	337 (46.7%)		
**Dependent variables**					
Depressive symptom	1.81 (0.46)	1.74 (0.49)	1.88 (0.39)	4.39	0.00
**Independent variables**					
physical victimization	1.81 (0.69)	1.77 (0.75)	1.84 (0.61)	1.45	0.15
relational victimization	1.39 (0.50)	1.36 (0.55)	1.42 (0.45)	1.79	0.07
verbal victimization	1.66 (0.64)	1.60 (0.69)	1.73 (0.56)	2.76	0.01
property victimization	1.49 (0.61)	1.43 (0.62)	1.57 (0.61)	2.94	0.00
social resources	3.45 (0.79)	3.60 (0.86)	3.26 (0.65)	-8.57	0.00
personal assets	3.54 (0.69)	3.74 (0.75)	3.32 (0.53)	-6.00	0.00


Pearson correlation coefficients among all observed variables are shown in **Table 2.** Peer victimization was positively associated with depressive symptoms but negatively associated with resilience. Furthermore, depressive symptoms and resilience were negatively correlated. All correlation coefficients were statistically significant at the *p* < 0.001 level.

**Table 2:** Correlation coefficients among observed variables.

	1	2	3	4	5	6	7	8	9
1 Physical victimization	1								
2 Relational victimization	0.42	1							
3 Verbal victimization	0.57	0.69	1						
4 Property victimization	0.45	0.54	0.57	1					
5 Personal assets	-0.21	-0.24	-0.25	-0.21	1				
6 Social resource	-0.33	-0.37	-0.42	-0.30	0.56	1			
7 Depressive factor1	0.33	0.38	0.41	0.31	-0.47	-0.54	1		
8 Depressive factor2	0.26	0.40	0.40	0.30	-0.34	-0.45	0.60	1	
9 Depressive factor3	0.30	0.42	0.41	0.30	-0.45	-0.50	0.70	0.64	1


*Depressive factor 1- Depressive factor 3 = three parcels of depressive symptoms. All bivariate coefficients are statistically significant at 0.001 level.*

### Direct Effects of Peer Victimization and Resilience on Depressive Symptoms

**FIGURE 1:**
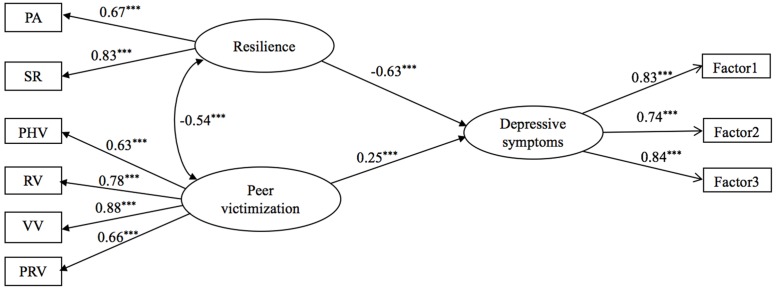
**Direct effects of peer victimization and resilience on depressive symptoms.** ^∗∗∗^*p* < 0.001.

Model 1 produced estimates of factor loadings and error variances for linear latent variables without an interaction term (**Figure 1**).

Net of demographic background (ie., gender and grade), both resilience and peer victimization were associated with depressive symptoms. In addition, peer victimization was positively linked to depressive symptoms (*β* = 0.25, *p* < 0.001), whereas resilience was a negative covariate of depressive symptoms (*β* = -0.63, *p* < 0.001). The model provided a good fit to the data: *χ^2^*/*df* = 3.05, TLI = 0.97, CFI = 0.98, RMSEA = 0.053. A total of about 62% of the variance of depressive symptoms was explained by the variables. The residual variance amounted to 0.38.

### Interaction Effects of Resilience and Peer Victimization on Depressive Symptoms

**FIGURE 2:**
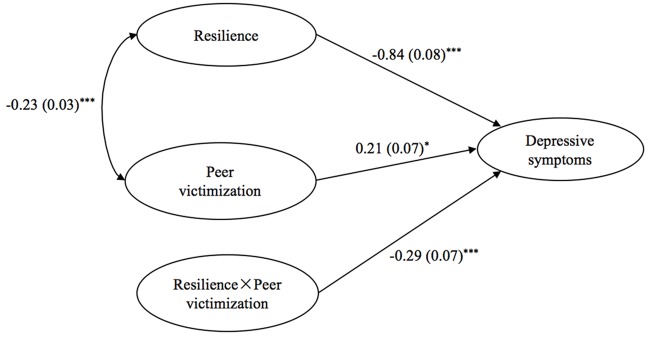
**Latent variable interaction model of peer victimization, resilience and depression.** ^∗^*p* < 0.05; ^∗∗∗^*p* < 0.001.

In Model 2, we added an interaction term as Klein and Moosbrugger (2000) suggested (**Figure 2**). In this model, peer victimization was significantly and positively associated with depressive symptoms (*B* = 0.21, *SE* = 0.07, *p* < 0.001), whereas resilience was significantly negatively associated with depressive symptoms (*B* = -0.84, *SE* = 0.08, *p* < 0.001). The model demonstrated a significant interaction effect of peer victimization and resilience on depressive symptoms (*B* = -0.29, *SE* = 0.07, *p* < 0.001). In terms of variance explained, the residual variance was slightly smaller in Model 2 (0.22) than in Model 1 (0.38).

**FIGURE 3:**
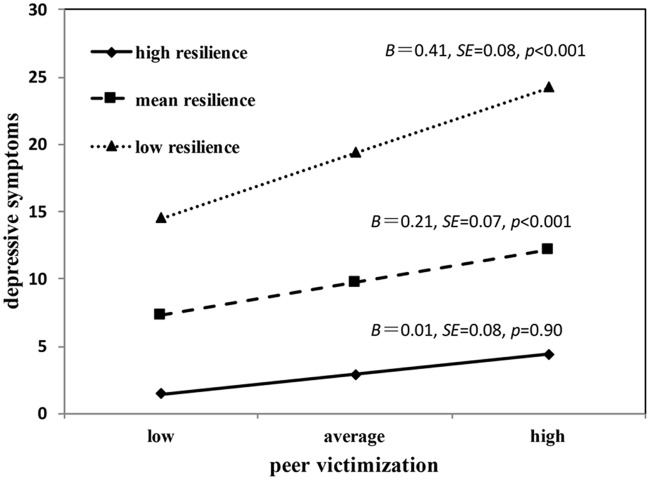
**Simple slope pattern for the interaction of peer victimization and resilience on depressive symptoms at low, average and high levels of resilience**.

To facilitate the interpretation of the interaction effects, we computed the simple slope of the regression of peer victimization on depressive symptoms at low (i.e., one *SD* below the mean), average, and high (i.e., one *SD* above the mean) values of resilience (**Figure 3**). Simple slope analysis revealed that the effect of peer victimization on depressive symptoms was positive and statistically significant at low levels of resilience (*B* = 0.41, *SE* = 0.08, *p* < 0.001) and average levels of resilience (*B* = 0.21, *SE* = 0.07, *p* < 0.001). However, at high levels of resilience, the association between peer victimization and depressive symptoms was non-significant (*B* = 0.01, *SE* = 0.08, *p* = 0.90).

### Multiple Group Analysis

Lastly, we used multiple group analysis to examine whether the interaction model differed significantly between the two groups of migrant children (i.e., private migrant school vs. public school). We first compared a model in which all structural paths were freely estimated with a model in which all structural paths were constrained to be equal. Constraining the model resulted in a statistically significant worsening of overall model fit (Δχ^2^ (3) = 12.88, *p* < 0.01). Therefore, we compared a model in which all structural paths were freely estimated between the two groups with a model in which we constrained only one of the three structural paths (i.e., two main effects, one interaction effect). The results indicated that the main effects of peer victimization (Δχ^2^ (1) = 6.54, *p* < 0.05) and resilience (Δχ^2^ (1) = 6.66, *p* < 0.01) on depressive symptoms were significantly different for the two groups. Specifically, children enrolled in migrant schools displayed a significantly stronger association between peer victimization and depressive symptoms (*B* = 0.47, *SE* = 0.15, *p* < 0.001) than children enrolled in traditional public schools (*B* = 0.09, *SE* = 0.11, *p* = 0.39). Furthermore, resilience had a significantly weaker predictive effect on depressive symptoms for children enrolled in migrant schools (*B* = -0.67, *SE* = 0.16, *p* < 0.001) than for children enrolled in traditional public schools (*B* = -2.41, *SE* = 0.84, *p* < 0.01). However, When we compared the unconstrained model and a model in which only the path pertaining to the interaction effect was constrained, we found no significant difference between the two models [Δχ^2^ (1) = 0.77, *p* = 0.38], indicating the interaction effect did not differ by children group.

## Discussion

The goal of this study was to examine the relationship between peer victimization and depressive symptoms among rural-to-urban migrant Chinese children and to investigate the role of resilience in modifying this relationship. First of all, our findings indicate that peer victimization is directly associated with depressive symptoms—increases in harassment, bullying, and other forms of peer victimization were linked with a greater number of symptoms of depression. This finding is consistent with previous studies (Ji et al., 2011; Zhang et al., 2009; Garnefski and Kraaij, 2014) but extends the link between peer victimization and depression to a new population—rural-to-urban Chinese migrant children. While all children encounter periods of transition, migrant children face particular psychosocial challenges as they adjust to the loss of one community while experiencing rapid immersion into another. As “outsiders” within their new social environments, rural-to-urban migrant children report high rates of peer victimization, and these victimization experiences should be taken seriously given the wide range of negative effects of bullying (Lee, 2011). Parents, caregivers, and educators should be aware of the increased risk for victimization and be vigilant for signs of depression, including sadness, social withdrawal, and changes in mood, appetite, and sleep (American Psychiatric Association, 2013). Our findings also suggest that links between victimization and depression are evident in early adolescence for this population. Peer relationships take on increased importance during this period, and peer victimization may be particularly detrimental to psychological health at this time (Chen et al., 2009; Wang and Mesman, 2015; Hamilton et al., 2016).

Secondly, our findings suggest that resilience moderates the association between peer victimization and depressive symptoms for rural-to-urban Chinese migrant children, such that the negative effects of peer victimization on depressive symptoms are reduced as resilience increases. Resilience may play a vital role in helping bullied children reduce their risk for depression and maintain psychosocial wellbeing. The results provide support for a *protective factor model* of resilience that suggests that resilience factors operate in an interactive and synergistic fashion to moderate the negative effects of risks (Fergus and Zimmerman, 2005; Zimmerman et al., 2013). Children who have strong external support networks, are able to regulate their emotions, and are competent problem solvers may fare better in the face of peer victimization (Lee and Cranford, 2008; Zhao and Zhao, 2015). Therefore, efforts to build strong support systems and improve coping skills for migrant children may be an effective way to counteract negative social experiences. Resilience-based interventions designed to improve the ability of migrant children to cope with daily challenges, manage negative cognitions, and maintain a hope for the future may be useful in equipping this population with the “ordinary magic” of resilience (Masten, 2001).

Finally, exploratory analyses suggest that the mechanisms impacting the relationships between peer victimization, depression, and resilience may differ depending upon school setting. The study’s sample consisted of rural-to-urban migrant children recruited from two settings: traditional public schools and a private migrant school. Findings suggest that the link between peer victimization and depressive symptoms was stronger for children enrolled in migrant schools, and that resilience provided weaker “defense” against depression for this group. In contrast, enrollment in a traditional public school seemed to offer a level of psychosocial protection for migrant children, even when accounting for demographic characteristics like socio-economic status. This is the second study to identify that public school attendance may actually serve a protective role for migrant children. Gao et al. (2015) found that despite concerns that attending school with non-migrant children could increase opportunity for victimization, public school enrollment for migrant children was actually linked with improved mental health across a wide variety of psychological outcomes. Several mechanisms could account for this, including characteristics of the school and impacts on the acculturation process. School climate has been found to have a major impact on children’s mental health (Thapa et al., 2012), and characteristics of Chinese public schools, including their superior infrastructure, greater financial support, strong faculty training, low teacher turnover, and comprehensive curricula may provide a better climate for vulnerable students. Secondly, immersion in the public schools may speed the acculturation process for newly arrived migrant children. Public schools offer better educational resources and more opportunities to connect with the host culture, which may in turn promote children’s social adaptation (Gao et al., 2015). In contrast, migrant schools are usually located on the outskirts of cities and served by rural, poorly qualified teachers, limiting the ability of migrant children to interact with people from their new city (Wang, 2008). Given the likelihood of migrant children to also reside in isolated, segregated migrant communities, public schools may be a key element in facilitating a healthy transition to the new urban environment.

China’s urban, informal migrant school system has exploded in the past decade, and research is critically needed to explore the impacts—both academic and psychosocial—of school setting for this vulnerable population. Our analysis suggests that the migrant school experience may increase psychosocial risk, with migrant school attendees reported higher levels of victimization, more symptoms of depression, and lower levels of resilience than migrant children enrolled in traditional public schools. The experience of attending a separate school for new migrants to Beijing may highlight the social vulnerability of this population and contribute to feelings of isolation and “otherness.” Future comparative studies are needed to explore the impact of the migrant school experience on psychosocial wellbeing and identify opportunities to improve the psychological health of the children who are relegated to this alternative, inequitable educational experience.

Future studies are also needed to tease apart resilience as a broad construct in order to identify sub-factors that may particularly effective in promoting positive outcomes for these youth, including close connections with others. According to Bronfenbrenner (1979), interpersonal relationships are the most important source of influence within a child’s immediate environment (i.e., the microsystem). Thus relationships with parents, teachers, and peers yield significant power to promote or hinder a child’s socio-emotional development and may offer promise in reducing risk and promoting positive outcomes for this population.

There are several limitations to the current study that warrant discussion. First, the sample consisted of migrant children from one Beijing district, thus limiting the ability of findings to generalize to migrants from other areas. Future studies could also be improved by recruiting a sample of non-migrant children who have moved within an urban area to serve as a comparison group so that the unique effects of the migrant acculturation experience could be better understood (Tucker and Long, 1998; Temple and Reynolds, 1999). Findings could also be strengthened if future studies evaluate key variables through additional methods besides self-report, including parent and teacher evaluations and/or direct observations of child behavior.

Despite these limitations, our findings are informative on at least three levels. First, employing a latent variable interaction model helps to overcome measurement error problems inherent in non-experimental data and thus produces a richer picture of the interactive nature of resilience than traditional statistical approaches (Trautwein et al., 2012; Hukkelberg et al., 2014). The study also provides information on the relationship between peer victimization and depressive symptoms and supports the development of resilience-focused interventions for children who are at-risk of peer victimization. Finally, the study contributes to an emerging body of literature on the psychosocial impact of the rural-to-urban migration experience of children in China and highlights the need for additional knowledge and resources to support this vulnerable population.

## Author Contributions

ZY wrote the first draft of the manuscript and assisted in study design, data collection, and data analyses. LC helped design the study and oversaw data collection. SH drafted the work and revised it critically for important intellectual content. HG assisted in study design and oversaw statistical analysis. XL revised the work critically for important intellectual content and assisted in study design. DL was the principal investigator of the study and led the study. All of the authors participated in the final approval of the version to be published and agreed to be accountable for all aspects of the work.

## Conflict of Interest Statement

The authors declare that the research was conducted in the absence of any commercial or financial relationships that could be construed as a potential conflict of interest.
